# Supplementary material for: Engagement with daily testing instead of self-isolating in contacts of confirmed cases of SARS-CoV-2
Source: BMC Public Health. 2021 Jun 5;21:1067. doi: 10.1186/s12889-021-11135-7 (PMC8178661; doi:10.1186/s12889-021-11135-7)
Supplement: Supplementary file 1 — Additional file 1. [file 12889_2021_11135_MOESM1_ESM.docx]

Supplementary materials

Supplementary table 1. Survey questions and answers

**Quarantine/testing evaluation questionnaire**

Thank you so much for taking part in this study of the views of quarantine and testing.

It would be very helpful if you could complete this brief survey about your views, so we can improve the service.

The survey only takes around 10-15 minutes to complete and it is anonymous - you do not need to give your name or any details about yourself.

Many thanks for sharing your views with us

The COVID Study Team

---------------------------------------------------------------------------------------------------------------------------

Please complete this questionnaire in one sitting

Use the arrows to go through the survey. The reset option clears answers from the current page.

To submit your answers click 'Submit' on the final page.

---------------------------------------------------------------------------------------------------------------------------

**The COVID study team.**

1. If you have been in contact with someone testing positive for coronavirus the usual option is to self-isolate by staying at home for 10 days.

A new option is to carry out daily home tests for up to 7 days, which means that every day you have a negative test you can carry on with your normal activities and do not need to self-isolate.

Which option do you prefer?

- Strongly prefer 10 day self-isolation option
- Somewhat prefer 10 day self-isolation option
- No preference for either option
- Somewhat prefer daily testing option
- Strongly prefer daily testing option
- Don't know

1. **If you had a positive test in the future and you knew that your contacts would be able to have daily testing (instead of self-isolating), would you be more or less likely to give their contact details?**

- Much more likely
- Somewhat more likely
- It would make no difference
- Somewhat less likely
- Much less likely
- Don't know

1. **Some people are being offered a daily testing kit by NHS Test and Trace. Did NHS Test and Trace offer a daily testing kit to you?**

- Yes, and I agreed that they could send one to me - go to Q4
- Yes, but I did not agree that they could send one to me – go to Q5
- No, I was not offered a daily testing kit – go to Q15

1. **Did you receive a testing kit in time for you to complete some daily testing rather than self-isolation?**

- Yes – go to Q6
- No - go to Q5

1. **Why did you decide not to take part in daily testing?**

**(tick all reasons that you agree with)**

- I don’t believe in the threat of coronavirus
- Daily testing seemed too difficult
- I felt the testing process could be unpleasant
- I don’t want to share data about myself with government bodies
- I feel unsure about the accuracy of the test results
- I couldn’t afford to have time off work if I tested positive
- I am probably immune, as I have already had coronavirus
- I don’t mix enough with others to be a risk to them
- It is easy, or easier for me to self-isolate
- I had symptoms, so I wanted to just take the usual test and self-isolate right away
- I believed I would test positive at some point, so it was easier to self-isolate from start
- I don't want to take any risk of spreading the virus to others
- I had already tested positive
- None of these reasons
- Other reason

On all answers move to Q15

1. **Why did you decide to complete daily tests rather than self-isolate for 10 days? (tick all reasons that you agree with)**

- I felt it was compulsory / I had no choice
- It sounded quite easy to do
- It would be difficult to self-isolate/ did not want to self-isolate
- I needed to go out to work
- My friends and family wanted me to do it
- My employer wanted me to do it
- I wanted to know if I had the virus
- I wanted to meet up with friends/family
- I know other people who were getting tested
- To help beat the virus in my area
- I was concerned that I might have coronavirus
- I needed to know if I was infected so I could protect vulnerable people that I live with or meet regularly
- None of these reasons
- Other reason

1. **How confident, if at all, are you that you did the tests correctly?**

- Completely confident
- Very confident
- Fairly confident
- Not very confident
- Not at all confident
- Don't know

1. **How confident, if at all, are you that your test results were accurate?**

- Completely confident
- Very confident
- Fairly confident
- Not very confident
- Not at all confident
- Don't know

1. **Did you have to repeat any home tests?**

**e.g. due to an inconclusive result or invalid test**

- Yes
- No

1. **Did any of these problems make it hard for you to do the tests and submit your results?**

**(tick all reasons that you agree with)**

- Instructions not clear
- I was not sure how to take the tests
- The testing procedure was unpleasant
- Daily testing was too much hassle
- I forgot to take one or more tests
- Internet / technology access
- I did not have any problems
- Don't know
- Other

1. **Starting from the day you first spoke to NHS Test and Trace, please tick the option that best describes the result of your daily test.**

**Please select an answer for each day.**

|  | **Positive** | **Negative** | **Inconclusive** | **I don’t know** | **I didn’t do a test** |
| --- | --- | --- | --- | --- | --- |
| **1** |  |  |  |  |  |
| **2** |  |  |  |  |  |
| **3** |  |  |  |  |  |
| **4** |  |  |  |  |  |
| **5** |  |  |  |  |  |
| **6** |  |  |  |  |  |
| **7** |  |  |  |  |  |

1. **On the days that you had a negative test result, did you do any of the following**

**(tick all reasons that you agree with)?**

- Go to the shops for groceries, toiletries, medicines or other items
- Attend a medical appointment (e.g. an outpatient appointment)
- Go to the shops for non-essential items
- Go to work, school or university
- Give help to, or provide care for, someone else (e.g. delivering food to them)
- Take a child to or from school
- Go for outdoor exercise (e.g. for a walk or run)
- Spend time indoors and in close contact (less than a meter apart and for more than 15 minutes) with friends or family you do not live with
- Go out for a meal or to an entertainment venue
- Go out for any other reason
- I did not do any activities

1. **Thinking about the days you had a negative result, did you have more or less close contact with people you do not live with (indoors and for more than 15 minutes) than you had before you were contacted by NHS Test and Trace?**

- Much more contact
- Slightly more contact
- About the same
- Slightly less contact
- Much less contact
- Does not apply

1. **Thinking about the days you did not take a test or had a positive/inconclusive test result, did you have more or less close contact with people you do not live with (indoors and for more than 15 minutes) than you had before you were contacted by NHS Test and Trace?**

- Much more contact
- Slightly more contact
- About the same
- Slightly less contact
- Much less contact
- Does not apply

1. **Thinking about the whole period from when you spoke to NHS Test and Trace to now, how many days, if any, did you self-isolate for? Please include any days before the daily tests arrived.**

- 1 – 14 days

1. **Why did you self-isolate?**

**(tick all reasons that you agree with)**

- I was complying with Government guidelines
- I was waiting for my test kit to arrive
- I performed daily testing and had a positive/inconclusive result
- I had symptoms
- Someone in my household had symptoms/ tested positive
- I stopped taking the daily tests or skipped some tests
- None of the above
- Other reason

1. **Starting from the time you first spoke to NHS Test and Trace, how often did you come into close contact (indoors and for more than 15 minutes) with someone that you do not live with?**

**For example, selecting the option 0 times would mean that you had no close contact, indoors and for more than 15 minutes, with anyone that you do not live with on that day.**

|  | **0 times** | **1 time** | **2-4 times** | **5-10 times** | **11 times or more** |
| --- | --- | --- | --- | --- | --- |
| **1** |  |  |  |  |  |
| **2** |  |  |  |  |  |
| **3** |  |  |  |  |  |
| **4** |  |  |  |  |  |
| **5** |  |  |  |  |  |
| **6** |  |  |  |  |  |
| **7** |  |  |  |  |  |

1. **On the days that you were trying to self-isolate, did you do any of the following**

**(tick all reasons that you agree with)?**

- Go to the shops for groceries, toiletries, medicines or other essential items
- Attend a medical appointment (e.g. an outpatient appointment)
- Go to the shops for non-essential items
- Go to work, school or university
- Give help to, or provide care for, someone else (e.g. delivering food to them)
- Take a child to or from school
- Go for outdoor exercise (e.g. for a walk or run)
- Spend time indoors and in close contact (less than a meter apart and for more than 15 minutes) with friends or family you do not live with
- Go out for a meal or to an entertainment venue
- Go out for any other reason
- I did not do any activities

1. **Please provide your age (optional)**
2. **Please provide your ethnicity (optional)**

- Asian - Indian
- Asian - Pakistani
- Asian - Bangladeshi
- Asian - Chinese
- Asian - Other
- Black - African
- Black - Caribbean
- Black - Other
- White British
- White Irish
- White - Other
- Mixed - White and Black Caribbean
- Mixed - White and Black African
- Mixed - White and Asian
- Mixed - Other
- Other ethnic group

1. **Please state when you left full time education**

**(if you are still in education, please select the stage you are at now)**

- Before finishing school
- After finishing school
- After finishing university
- After finishing post graduate studies

1. **Please provide your name (optional)**
2. **Please provide the ID provided by NHS Test and Trace (optional)**
3. **Are there any other comments or suggestions you have to improve the daily self-testing service?**

----------------------------------------------------------------------------------------------------------------------

Thank you for taking the time to complete this survey.

Your responses will help to improve the NHS Test and Trace service.

For more information about COVID-19, please visit: <https://www.gov.uk/coronavirus>

Supplementary table 2. Issues with daily testing

|  | N | % |  |
| --- | --- | --- | --- |
| Instructions not clear | 12 | 4% |  |
| I was not sure how to take the tests | 1 | 0% |  |
| The testing procedure was unpleasant | 50 | 15% |  |
| Daily testing was too much hassle | 4 | 1% |  |
| I forgot to take one or more tests | 2 | 1% |  |
| Internet / technology access | 18 | 6% |  |
| I did not have any problems | 220 | 67% |  |
| Don't know | 3 | 1% |  |
| Other | 16 | 5% |  |
| *Item: "Did any of these problems make it hard for you to do the tests and submit your results?..."* | | |  |
|  |  |  |  |

Supplementary table 3. Why did you decide to complete daily tests rather than self-isolate for 10 days?

| Item | Whole sample | % | Ethnic minority | % | White | % | Secondary education | % | Higher education | % |  |
| --- | --- | --- | --- | --- | --- | --- | --- | --- | --- | --- | --- |
| I felt it was compulsory / I had no choice | 3 | 0% | 2 | 3% | 1 | 0% | 1 | 0% | 1 | 0% |  |
| It sounded quite easy to do | 175 | 17% | 13 | 16% | 151 | 17% | 54 | 16% | 97 | 17% |  |
| It would be difficult to self-isolate/ did not want to self-isolate | 81 | 8% | 5 | 6% | 72 | 8% | 36 | 10% | 41 | 7% |  |
| I needed to go out to work | 38 | 4% | 5 | 6% | 31 | 3% | 8 | 2% | 26 | 5% |  |
| My friends and family wanted me to do it | 16 | 2% | 2 | 3% | 14 | 2% | 7 | 2% | 6 | 1% |  |
| My employer wanted me to do it | 3 | 0% | 0 | 0% | 2 | 0% | 2 | 1% | 1 | 0% |  |
| I wanted to know if I had the virus | 226 | 22% | 15 | 19% | 195 | 22% | 80 | 23% | 122 | 22% |  |
| I wanted to meet up with friends/family | 21 | 2% | 0 | 0% | 21 | 2% | 7 | 2% | 11 | 2% |  |
| I know other people who were getting tested | 10 | 1% | 3 | 4% | 7 | 1% | 5 | 1% | 4 | 1% |  |
| To help beat the virus in my area | 206 | 20% | 11 | 14% | 180 | 20% | 66 | 19% | 117 | 21% |  |
| I was concerned that I might have coronavirus | 91 | 9% | 9 | 11% | 76 | 9% | 32 | 9% | 46 | 8% |  |
| I needed to know if I was infected so I could protect vulnerable people that I live with or meet regularly | 133 | 13% | 11 | 14% | 113 | 13% | 38 | 11% | 73 | 13% |  |
| None of these reasons | 2 | 0% | 0 | 0% | 1 | 0% | 0 | 0% | 2 | 0% |  |
| Other reason | 28 | 3% | 3 | 4% | 25 | 3% | 9 | 3% | 13 | 2% |  |
| *Item: "Why did you decide to complete daily tests rather than self-isolate for 10 days?..."* | | | | | | | | | | |  |
| *Note. This item was also asked elsewhere in the participant recruitment that was not part of this survey, so numbers reported elsewhere may differ* | | | | | | | | | | |  |
|  |  |  |  |  |  |  |  |  |  |  |  |
